# Supplementary material for: Out-of-Plane Biphilic Surface Structuring for Enhanced Capillary-Driven Dropwise Condensation
Source: Langmuir. 2023 Jan 16;39(4):1585–92. doi: 10.1021/acs.langmuir.2c03029 (PMC9893811; doi:10.1021/acs.langmuir.2c03029)
Supplement: Supplementary file 1 — la2c03029_si_001.pdf [file la2c03029_si_001.pdf]

Supporting Information

Out-of-Plane Biphilic Surface Structuring for Enhanced Capillary-Driven Dropwise Condensation

Luca Stendardo[1,†], Athanasios Milionis[1], George Kokkoris[2,3‡], Christos Stamatopoulos[1], Chander Shekhar Sharma[4], Raushan Kumar[4], Matteo Donati[1], Dimos Poulikakos[1,]*

[1]Laboratory of Thermodynamics in Emerging Technologies (LTNT), Sonneggstrasse 3, ETH Zurich, 8092 Zurich, Switzerland

[2]Institute of Nanoscience and Nanotechnology, NCSR Demokritos, Agia Paraskevi 15341, Greece

[3] School of Chemical Engineering, National Technical University of Athens, Heroon Polytechniou 9, Zografou, Athens 15780, Greece

[4]Thermofluidics Research Lab, Department of Mechanical Engineering, Indian Institute of Technology Ropar, Rupnagar, Punjab, 140001 India

This supporting information document contains detailed descriptions of the user-defined mass transfer model, the conservation laws and the VOF method, the dynamic contact angle model, the droplet jumping velocity on biphilic and superhydrophobic micro-cavities, and the mesh independence study.

TABLE OF CONTENT

Section S1. User-defined mass transfer model…………………………………………… S2

Section S2. Conservation laws and VOF method……………………………………... S4

Section S3. Dynamic contact angle model……………………………………………….. S5

Section S4. Jumping velocity on biphilic and superhydrophobic micro-cavities………... S7

Section S5. Mesh independence study…………………………………………………… S8

SECTION S1. USER-DEFINED MASS TRANSFER MODEL

$$R = R_0 t^a \quad \text{(S1)}$$

$$\frac{1}{V}\frac{dV}{dt} = \mathrm{m}t^a \quad \text{(S2)}$$

$$\frac{1}{V}\frac{dm}{dt} = \rho \mathrm{m} \mathrm{t}^{\mathrm{a}} \quad \text{(S3)}$$

where m and R_0 are constants, t is the flow time and a represents the flow time exponent ($a < 1$).

The equations presented (S1 and S2) describe the mass transfer model[1] that has been used as an alternative to the Lee[2] model (conventionally used for phase change modeling)[3] during the simulations on the textured surfaces. The equations are derived by considering the cell source ($m\ t^a$) times the total volume (V) as the time derivative of the volume (equation S2). This model allows to "add liquid" to a small droplet placed as an initial condition at the base of the micro-cavity, while excluding the formation of other droplets due to the vapor to liquid phase transition predicted by the Lee model. The model works by defining a mass source of liquid water in every cell, where the liquid fraction α is equal to 1. This means that all the computational cells inside the droplet are automatically defined as mass sources, adding new liquid to the droplet over time. In this way the water mass is added without any momentum. The advantage of this mass transfer model is that it automatically "follows" the droplet and at every instant water is added to the droplet from inside and not by an external source. This assures that the droplet motion is not influenced by the addition of mass but only by the constriction that the texture walls exert on the growing droplet. For this mass transfer model, the energy equation is not solved (see section S2) and the mass addition depends only on the flow time. To optimize computational time, $m = 1100$ and $a = -1.2$.

To check that the growth exponent a does not influence the droplet's surface interactions and, in general, the droplet mobility, a control run with $a = -0.6$ has been compared to a simulation run with $a = -1.2$ on a domain with four pillar elements. The droplet grew at the centre of the domain in both cases and attention has been given to the pillar squeezing effect and to the subsequent jumping of the droplet.

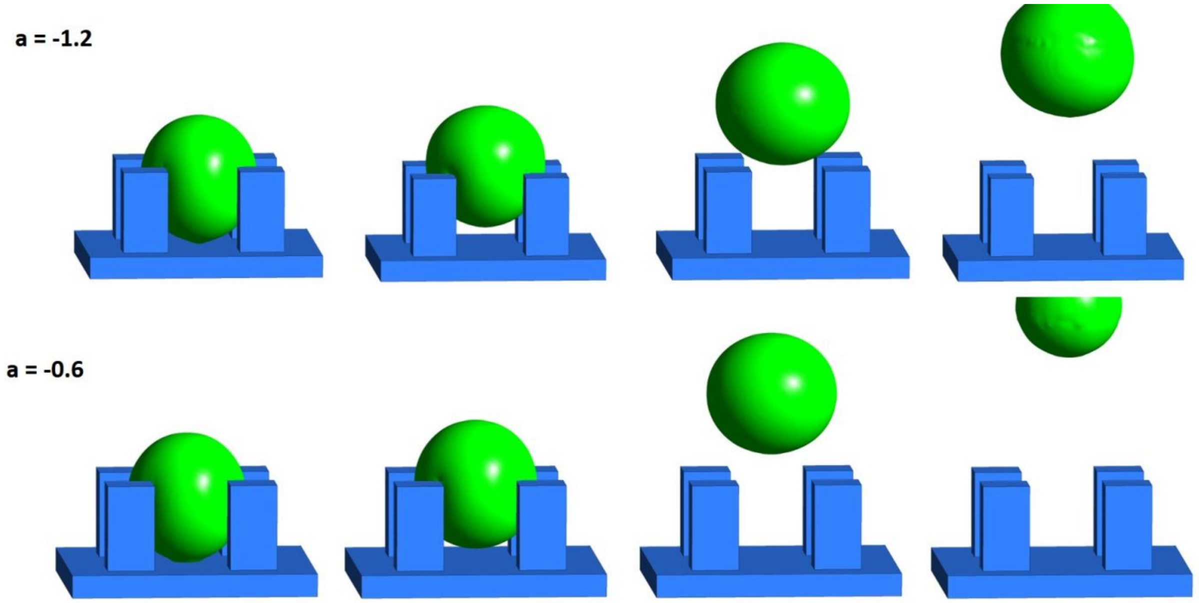

Figure S 1. Comparison between different volume growth exponents, respectively a = -1.2 and a = -0.6. The droplet grew on a pillar domain.

Figure S1 shows two cases, identical except for the value of exponent a, of a droplet growing between four straight-walled pillars. As can be seen from the images, the behaviour of the droplet

was qualitatively identical. The droplet detachment volumes were 3.44×10^{-13} m^3 for $a = -1.2$ and 3.17×10^{-13} m^3 for $a = -0.6$, therefore the difference amounts to less than 10%. The case with $a = -1.2$ required approximately 3 times lower number of time-steps compared to the case $a = -0.6$ (and thus also 3 times less computational time).

SECTION S2. CONSERVATION LAWS AND VOF METHOD

The Volume of Fluid (VOF)[3,4] method is based on a scalar field that represents the portion of phase volume occupation for every computational cell. The volume fractions of liquid (l) and vapour phase (v) are constrained to sum up to 1.

$$\alpha_l + \alpha_v = 1 \tag{S4}$$

The working fluid's properties, such as density ρ, dynamic viscosity μ, and thermal conductivity λ, are calculated by processing the volume fractions.

$$\begin{cases} \rho = \alpha_l \rho_l + \alpha_v \rho_v \\ \mu = \alpha_l \mu_l + \alpha_v \mu_v \\ \lambda = \alpha_l \lambda_l + \alpha_v \lambda_v \end{cases} \tag{S5}$$

The continuity equations in VOF are:[3]

$$\begin{cases} \frac{\partial \alpha_l}{\partial t} + \nabla \cdot (\vec{v} \alpha_l) = \frac{\dot{m}_{v \to l}}{\rho_l} \\ \frac{\partial \alpha_v}{\partial t} + \nabla \cdot (\vec{v} \alpha_v) = \frac{\dot{m}_{l \to v}}{\rho_v} \end{cases} \tag{S6}$$

where t represents time, $\vec{\boldsymbol{v}}$ is the velocity vector and $\dot{m}$ indicates a source term that considers the mass transfer rate through the two-phase interface. The momentum equation is written as:[3]

$$\frac{\partial}{\partial t}(\rho\vec{v}) + \nabla\cdot(\rho\vec{v}\vec{v}) = -\nabla p + \nabla\cdot\left[\mu(\nabla\vec{v} + \nabla\vec{v}^{T}) - \frac{2}{3}\mu\nabla\cdot\vec{v}\,I\right] + \rho\vec{g} + F_{vol} \qquad \text{(S7)}$$

where p represents the pressure, I is the unit matrix and $\vec{\boldsymbol{g}}$ is the gravitational acceleration. The Continuum Surface Force (CSF) model proposed by Brackbill et al.[5] has been implemented in the VOF model. In this way, the surface tension acts as a volume force and can be considered a source term in the momentum equation:[3,5]

$$F_{vol} = \sigma\frac{(\alpha_l\rho_l\kappa_v\nabla\alpha_v + \alpha_v\rho_v\kappa_l\nabla\alpha_l)}{\frac{1}{2}(\rho_l + \rho_v)} \qquad \text{(S8)}$$

The surface tension is therefore a continuous volume force spread across the interface region. Here, σ represents the interfacial tension force between liquid and gas phase, specified as 0.072 N/m in this work. The CSF model by Brackbill et al.[5] is used as well to compute the surface curvature and this model takes into account the local volume fraction gradients normal to the surface:[3,5]

$$\begin{cases} \kappa_l = \nabla\cdot\frac{\nabla\alpha_l}{|\nabla\alpha_l|} \\ \kappa_v = \nabla\cdot\frac{\nabla\alpha_v}{|\nabla\alpha_v|} \end{cases} \qquad \text{(S9)}$$

Imposing a contact angle at the liquid-solid interface, therefore intentionally changing the local curvature of the surface in the cells near the wall, influences the surface tension force term, which again modifies the momentum equation.

SECTION S3. DYNAMIC CONTACT ANGLE MODEL

A droplet in a dynamic state generally involves two contact angle values, an advancing, and a receding angle respectively. Therefore, the curvature of the droplet surface should vary depending on the dynamic condition of the droplet and on the instantaneous value of the contact angle at the droplet contact line. In Ansys Fluent, however, it is not possible to define an advancing and a receding contact angle to capture the exact motion of the droplet; instead, a static contact angle model is used by default. To overcome this limitation, a dynamic contact angle model based on the empirical correlation of Kistler[6] has been programmed through a User Defined Function and included in the simulation framework. This particular model has been used in various previous studies of droplet dynamics[7–9] and can be considered appropriate for this study since the droplet contact line generally moves from an equilibrium state (of the sessile droplet) on a partially wetted

surface. This would not be the case for droplet impact onto dry surfaces for example, where the contact angle assumes values up to 180° initially.

The contact angle at the liquid-solid interface is given by:[6,10]

$$\theta = f_H\left[Ca + f_H^{-1}(\Theta)\right] \tag{S10}$$

where Ca is the contact line capillary number and f_H is the Hoffman function,

$$f_H(s) = \arccos\left\{1 - 2\tanh\left[5.16\left(\frac{s}{1 + 1.31 s^{0.99}}\right)^{0.706}\right]\right\} \tag{S11}$$

The capillary number is given by

$$Ca = \mu u_{cl}/\sigma \tag{S12}$$

where u_{cl} is the contact line velocity. The dummy variable Θ is used to capture contact angle hysteresis and its value depends on the direction of the contact line velocity:

$$\Theta = \begin{cases} \theta_a & for\ u_{cl} > 0 \\ \theta_0 & for\ u_{cl} = 0 \\ \theta_r & for\ u_{cl} < 0 \end{cases} \tag{S13}$$

where θ_a, θ_0 and θ_r are the imposed advancing, static, and receding contact angles respectively.

SECTION S4. JUMPING VELOCITY ON BIPHILIC AND SUPERHYDROPHOBIC MICRO-CAVITIES

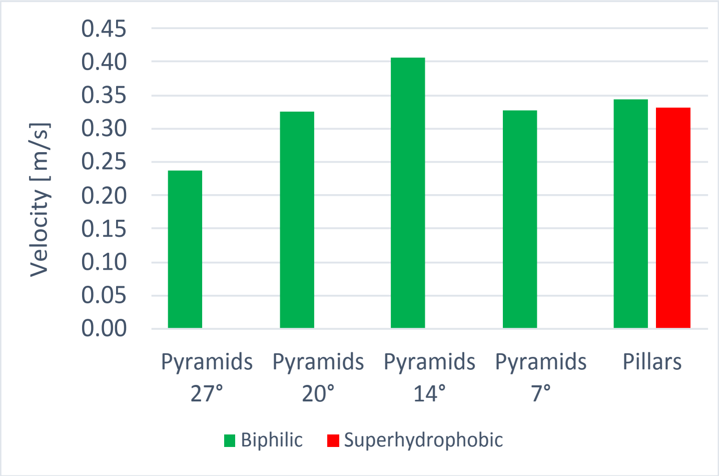

Figure S2. Surface clearing jumping velocity measured for all micro-geometries. The hydrophilic spot causes jumping droplets on all five geometries.

Figure S2 shows the jumping velocity out of the microcavity for all five considered micro-texture geometries. The hydrophilic spot induces a jumping event on all domains, regardless of the micro-texture geometry. The jumping velocity is lies between 0.20 m/s and 0.40 m/s for the various geometries. Without a hydrophilic spot, only the pillars can cause a surface clearing jumping event.

SECTION S5. MESH INDEPENDENCE STUDY

A mesh independence study with coarse, medium, fine, and the finest mesh was conducted, which had 142’352, 554’840, 1’056’000, and 2’477’538 mesh elements respectively. For this study, the droplet dynamics were investigated by tracking the droplet interface throughout a coalescence and subsequent jumping event. The simulation case that was chosen for this study is the one presented in Figure 4b of the main text: two droplets growing in a pillar domain with hydrophilic spot. At the moment of coalescence, tracking of the droplet interface started, recording the Y-position of the highest point of the coalescing droplet.

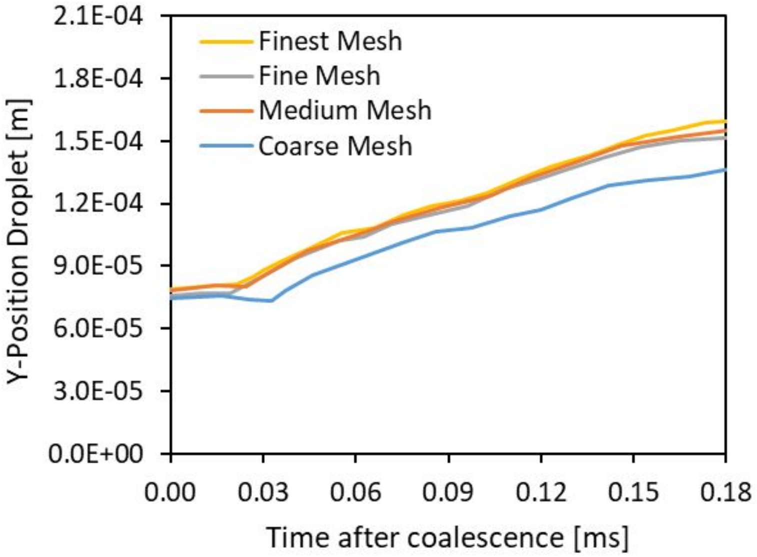

Figure S3. Mesh independence study with comparison of the Y-position of the highest point of the droplet. The Y-position was measured as the absolute position inside the computational domain. The difference from the medium mesh to the finest mesh was measured to be less than 3%, thus mesh independence is established.

The change of the interface position from the medium mesh to the finest mesh was less than 3%, thus mesh independence was verified. The cell sizes of the meshes are 4 μm, 2.5 μm, 2 μm, and 1.5 μm respectively. For the simulations presented in this work, a mesh with average cell size of 2 μm was chosen.

AUTHOR INFORMATION

Corresponding Author

*E-mail: dimos.poulikakos@ethz.ch

Present Address

†Department of Materials Science, University of Milano – Bicocca, via R. Cozzi 55, 20125 Milano, Italy

‡ School of Chemical Engineering, National Technical University of Athens, Heroon Polytechniou 9, Zografou, Athens 15780, Greece

AUTHOR CONTRIBUTIONS

The manuscript was written through the contributions of all authors. All authors have given approval to the final version of the manuscript.

NOTES

The authors declare no competing financial interest.

ACKNOWLEDGMENTS

This project received funding from the European Union's Horizon 2020 research and innovation program under grant number 801229 (‘HierARchical Multiscale NanoInterfaces for enhanced Condensation processes’ - HARMoNIC).

REFERENCES

(1) Sharma, C. S.; Combe, J.; Giger, M.; Emmerich, T.; Poulikakos, D. Growth Rates and Spontaneous Navigation of Condensate Droplets Through Randomly Structured Textures. *ACS Nano* **2017**, *11*, 1673–1682.

(2) Lee, W. H. A Pressure Iteration Scheme for Two-Phase Flow Modeling. *Tech. Rep. -UR N. M. Los Alamos Sci. Lab.* **1979**, 408–431.

(3) Ke, Z.; Shi, J.; Zhang, B.; Chen, C. Numerical Investigation of Condensation on Microstructured Surface with Wettability Patterns. *J Heat Transf* **2017**, *115*, 1161–1172.

(4) Ansys Fluent Theory Guide, Release 19.0, 2018.

(5) Brackbill, J. U.; Kothe, D. B.; Zemach, C. A Continuum Method for Modeling Surface Tension. *J. Comput. Phys.* **1992**, *100*, 335–354.

(6) Kistler, S. F. Hydrodynamics of Wetting. *J C Berg Ed Wettability* **1993**, 311.

(7) Roisman, I. V.; Opfer, L.; Tropea, C.; Raessi, M.; Mostaghimi, J.; Chandra, S. Drop Impact onto a Dry Surface: Role of the Dynamic Contact Angle. *Colloids Surf. Physiochemical Eng. Asp.* **2008**, *322*, 183–191.

(8) Mukherjee, S.; Abraham, J. Investigations of Drop Impact on Dry Walls with a Lattice-Boltzmann Model. *J. Colloid Interface Sci.* **2007**, *312* (2), 341–354. https://doi.org/10.1016/j.jcis.2007.03.004.

(9) Šikalo, Š.; Wilhelm, H.-D.; Roisman, I. V.; Jakirlić, S.; Tropea, C. Dynamic Contact Angle of Spreading Droplets: Experiments and Simulations. *Phys. Fluids* **2005**, *17* (6), 062103. https://doi.org/10.1063/1.1928828.

(10) Sykes, T. C.; Harbottle, D.; Khatir, Z.; Thompson, H. M.; Wilson, M. C. T. Substrate Wettability Influences Internal Jet Formation and Mixing during Droplet Coalescence. *Langmuir* **2020**, *36* (32), 9596–9607. https://doi.org/10.1021/acs.langmuir.0c01689.
